# Ketogenic diet–induced changes in methylation status and neuropeptide signaling: relationships between S-adenosylmethionine (AdoMet), orexin-A, and metabolic health

**DOI:** 10.3389/fphys.2025.1719549

**Published:** 2025-11-04

**Authors:** Laura Mosca, Antonietta Monda, Antonietta Messina, Francesca Cadoni, Fiorenzo Moscatelli, Marco La Marra, Vincenzo Monda, Girolamo Di Maio, Salvatore Allocca, Claudia Casella, Maria Casillo, Rita Polito, Marcellino Monda, Marina Porcelli, Giovanni Messina

**Affiliations:** ^1^ Department of Precision Medicine, University of Campania “Luigi Vanvitelli”, Naples, Italy; ^2^ Department of Human Science and Quality of Life Promotion, San Raffaele Telematic University, Rome, Italy; ^3^ Department of Education and Sport Sciences, Pegaso Telematic University, Naples, Italy; ^4^ Department of Experimental Medicine, Section of Human Physiology, Unit of Dietetics and Sports Medicine, University of Campania “Luigi Vanvitelli”, Naples, Italy; ^5^ Department of Economics, Law, Cybersecurity, and Sports Sciences, University of Naples “Parthenope”, Naples, Italy; ^6^ Department of Psychology and Health Sciences, Pegaso Telematic University, Naples, Italy; ^7^ Department of Advanced Biomedical Sciences – Section of Legal Medicine, University of Naples “Federico II”, Naples, Italy

**Keywords:** S-adenosylmethionine, orexin-A, ketogenic diet, neuropeptide signaling, methylation status

## Abstract

**Introduction:**

S-Adenosylmethionine (AdoMet) is the principal methyl donor in numerous biochemical reactions, regulating lipid and glucose metabolism, inflammatory pathways, and neurotransmitter synthesis. Orexin-A, a hypothalamic neuropeptide involved in arousal, feeding behavior, and energy expenditure, plays a crucial role in metabolic homeostasis. Emerging evidence suggests potential crosstalk between methylation pathways and orexinergic signaling; however, this interaction has not been explored in the context of nutritional ketosis. This study aimed to evaluate the effects of a structured ketogenic dietary intervention on circulating AdoMet, Orexin-A, and key metabolic parameters.

**Methods:**

Twenty-one adults (11 males, 10 females) were recruited from the Unit of Dietetics, Sports Medicine, and Psychophysical Wellbeing, University Hospital “L. Vanvitelli” of Naples, Italy. Participants followed a ketogenic diet for 8 weeks. Anthropometric measurements, body composition, fasting biochemical parameters, AdoMet, and Orexin-A levels were assessed at baseline (T0) and post-intervention (T1). Paired t-tests were used to analyze within-subject changes, and linear regression analyses explored correlations between AdoMet and metabolic variables. The study was approved by the Ethics Committee of the University of Campania “Luigi Vanvitelli“ (Protocol No. 0003232/I, 01/02/2023).

**Results:**

After 8 weeks, participants exhibited significant reductions in body weight, BMI, visceral adipose tissue, total cholesterol, fasting glycemia, triglycerides, and HbA1c (all p < 0.05). Circulating AdoMet (−75.7%) and Orexin-A (−7.2%) levels also decreased significantly. AdoMet levels positively correlated with body weight, BMI, visceral adipose tissue, total cholesterol, fasting glycemia, triglycerides, HbA1c, and notably Orexin-A (R^2^ = 0.3848, p < 0.0001).

**Discussion:**

The ketogenic diet significantly improved anthropometric and metabolic parameters while concurrently reducing AdoMet and Orexin-A levels. The strong positive correlation between AdoMet and Orexin-A suggests an interaction between methylation status and neuropeptide signaling during metabolic adaptation to ketosis. These findings highlight AdoMet as a potential integrated biomarker of metabolic health and emphasize the relevance of neuro-metabolic regulation in dietary interventions.

## Introduction

S-Adenosylmethionine (AdoMet also known as SAM or SAMe) is a pivotal metabolite in cellular physiology, functioning as the universal methyl donor for a wide range of biochemical reactions, including DNA and histone methylation, phospholipid synthesis, and hormone metabolism (Chiang et al., 1996; Mato et al., 2013; Capelo-Diz et al., 2023). Through these methylation-dependent processes, AdoMet exerts a regulatory influence over lipid and glucose homeostasis, inflammatory pathways, and neurotransmitter synthesis, thereby linking nutrient status to epigenetic and metabolic control (Pascale et al., 2022; Roberti et al., 2021). Due to its crucial role in multiple biochemical pathways, AdoMet has been intensively studied as a potential therapeutic agent for the treatment of various clinical disorders. AdoMet is an FDA-approved dietary supplement, and pharmaceutical preparations of this compound are available in intravenous, intramuscular, and oral forms (Mosca et al., 2022). The sulfonium compound is an effective anti-inflammatory, antidepressant and analgesic molecule used in the therapeutic treatment of depression, liver disorders, musculoskeletal and joint disorders such as, osteoarthritis, fibromyalgia, neurodegenerative disorders, and most notably, major depressive disorders (MDD) (Mosca et al., 2022). Reviews of clinical trials to date indicate that, at pharmacological doses, AdoMet has a low incidence of side effects with an excellent level of tolerability (Mosca et al., 2022). Dysregulation of AdoMet metabolism - whether due to altered dietary methionine intake, impaired one-carbon metabolism, or chronic metabolic stress - has been implicated in obesity, type 2 diabetes mellitus, and cardiovascular disease (Capelo-Diz et al., 2023; Poirier et al., 2001; Froese et al., 2019). Despite these associations, the potential of AdoMet as a sensitive and integrative biomarker of metabolic health, capable of reflecting changes across multiple physiological domains, remains underexplored in human studies.

Orexin-A, also known as hypocretin-1, is a neuropeptide produced in the lateral hypothalamus that plays a crucial role in the regulation of arousal, feeding behaviour, and energy expenditure. Beyond its central effects on sleep–wake regulation, orexin-A influences peripheral metabolic processes, including glucose utilization, lipid oxidation, and thermogenesis, positioning it as an important integrator of energy balance (Sakurai, 2003; Butterick et al., 2013). Emerging evidence suggests that orexinergic signaling may intersect with methylation pathways, as both systems respond to nutrient availability and metabolic stress. For example, changes in one-carbon metabolism can influence hypothalamic neuropeptide expression, while orexin activity itself can modulate metabolic efficiency and inflammatory tone. However, this potential crosstalk between AdoMet -dependent methylation status and orexinergic regulation has not yet been examined in the context of coordinated systemic metabolic changes (Guerrero et al., 2022).

The ketogenic diet—a high-fat, moderate-protein, and very low-carbohydrate dietary regimen—induces a metabolic shift from glucose to fatty acids and ketone bodies (β-hydroxybutyrate, acetoacetate) as the primary energy substrates (Fortier et al., 2021; Turetta et al., 2025; Leung et al., 2025). This metabolic adaptation is associated with improvements in body composition, lipid profile, glycemic control, and mitochondrial function (Hilary et al., 2024; Khodabakhshi et al., 2021). Moreover, ketogenic states have been shown to modulate both methylation pathways and orexinergic signaling, potentially providing a unifying mechanism for their beneficial effects on metabolic health (Dabek et al., 2020; Weinshenker, 2008).

In light of these considerations, the present study was designed to assess the impact of a structured ketogenic dietary intervention on circulating AdoMet levels, anthropometric indices, lipid and glucose metabolism, and plasma orexin-A concentrations.

## Materials and methods

### Study design and participants

This was a longitudinal observational study evaluating the effects of a structured lifestyle and metabolic intervention on anthropometric, biochemical, and neuroendocrine parameters. Participants were recruited from the Unit of Dietetics and Sports Medicine, University of Campania “Luigi Vanvitelli.” The study protocol was approved by the Ethics Committee of the University of Campania “Luigi Vanvitelli” (Protocol No. 0003232/I del 01/02/2023), and all procedures were conducted in accordance with the Declaration of Helsinki. Written informed consent was obtained from all participants prior to inclusion in the study. The final study sample consisted of 21 adults (11 males and 10 females) who met the eligibility criteria. Inclusion criteria were age between 18 and 65 years, absence of acute or chronic inflammatory diseases, stable medication regimen for at least 3 months, and willingness to comply with study procedures. Exclusion criteria included: pregnancy or lactation, severe hepatic or renal dysfunction, active cancer, or recent participation in another clinical trial. Participants underwent an 8-week isocaloric ketogenic diet designed to induce nutritional ketosis. The dietary composition consisted of approximately 43% of total calories from fat, 43% from protein, and 14% from carbohydrates (<50 g/day). Individual energy needs were calculated using the Harris–Benedict equation adjusted for physical activity level. The diet included healthy fat sources (olive oil, nuts, avocado, fatty fish), lean proteins, and low-carbohydrate vegetables. Adherence to the diet was supervised weekly by a dietitian, and nutritional ketosis was confirmed through capillary β-hydroxybutyrate (BHB) measurements (>0.5 mmol/L) and urinary ketone strips (Acetoacetate).

### Anthropometric and body composition measurements

Body weight and height were measured using a calibrated stadiometer and digital scale, with participants wearing light clothing and no shoes. Body mass index (BMI) was calculated as weight (kg) divided by height squared (m^2^). Visceral adipose tissue (VAT) mass was assessed using a validated multifrequency bioimpedance analyzer (BIA).

### Biochemical and hormonal measurements

Fasting venous blood samples were collected at baseline (T0) and after the intervention (T1). Serum samples were aliquoted and stored at −80 °C for up to 6 months before analysis. All samples underwent a single freeze–thaw cycle. AdoMet and Orexin-A concentrations were determined using validated ELISA kits [AdoMet: S-Adenosylmethionine (SAM) ELISA Kit, Cell Biolabs, INC.; Orexin-A: Human OXA Elabscience (E-EL-H1015)] according to manufacturer instructions. Each sample was analyzed in duplicate, and any pair with >15% coefficient of variation (CV) was re-run. Inter- and intra-assay CVs were below 10%. Calibration curves were generated using a 5-parameter logistic regression (*R* ^2^ > 0.99), and QC samples were included on each plate to ensure consistency across runs. For each assay (AdoMet and Orexin-A), calibration curves were fitted using a five-parameter logistic (5-PL) regression model as recommended by the ELISA manufacturers. The quantification range covered 0.5–100 ng/mL for AdoMet and 0.1–10 ng/mL for Orexin-A. The coefficient of determination (*R* ^2^) for all standard curves exceeded 0.99, indicating excellent curve fitting accuracy across the dynamic range. Representative standard curves, including residual distributions, are provided in Supplementary Figure S1.

All sample measurements fulfilled the assay quality acceptance criteria, with intra-assay and inter-assay CVs <10% and recovery within 90%–110% of expected values. Quality control (QC) samples were analyzed in each run to confirm plate-to-plate reproducibility. Total cholesterol, triglycerides, fasting plasma glucose, and HbA1c were measured using standard automated methods in the hospital’s central laboratory.

### Statistical analysis

All data were tabulated and expressed as mean ± standard deviation (SD). Statistical analyses were performed using GraphPad Prism version 9.5.1 (GraphPad Software, LLC, San Diego, CA, United States; 2023). Prior to performing any comparisons, the distribution of each variable was assessed using the Shapiro–Wilk test to evaluate the assumption of normality.

For variables that followed a normal distribution, parametric tests were applied. Specifically, paired t-tests were used to compare measurements before (T0) and after (T1) the intervention. In cases where data did not follow a normal distribution, appropriate non-parametric tests were employed. The level of statistical significance was set at *p* < 0.05. In addition to statistical significance testing, effect sizes were calculated to quantify the magnitude of changes observed between baseline (T0) and post-intervention (T1). For paired comparisons, Cohen’s *d* was computed as the mean of the paired differences divided by the standard deviation of the differences. Effect sizes were interpreted as small (*d* = 0.2–0.49), medium (*d* = 0.5–0.79), and large (*d* ≥ 0.8), following conventional thresholds. Effect size values are reported in Table 1 alongside the corresponding *p*-values.

**TABLE 1:** Paired t-test results comparing values at baseline (T0) and after the intervention period (T1).

Parameters	T0		T1		p	Choen’s d
AdoMet (pg/mL)	536.54	±248.91	130.21	±88,03	<0.001	2.18
Weight (kg)	93.95	±15.12	76.59	±14.53	<0.001	1.17
BMI (kg/m^2^)	31.65	±4.43	26.47	±3.66	<0.001	1.27
VAT (g)	1769.80	±751.11	902.76	±544.97	<0.001	1.32
Total cholesterol (mg/dL)	241.28	±29.69	184.38	±19.30	<0.001	2.27
Glycemia (mg/dL)	111.76	±7.97	87.04	±9.52	<0.001	2.82
Triglycerids (mg/dL)	176.21	±60.02	79.57	±32.90	<0.001	2.0
Hbac1 (%)	5.76	±0.42	5.53	±0.28	<0.05	0.64
Orexin-A (pg/mL)	3085.79	±87.25	2862.49	±68.44	<0.001	2.85

Data are expressed as mean ± standard deviation. All reported differences were statistically significant.

AdoMet, S-Adenosylmethionine; BMI, body mass index; VAT, visceral adipose tissue; HbA1c, Glycated Hemoglobin; T0, baseline; T1, Post-intervention.

In addition to group comparisons, simple linear regression analyses were conducted to evaluate the association between serum AdoMet levels and various anthropometric and metabolic parameters, including body weight, BMI, visceral adipose tissue (VAT), total cholesterol, fasting glycemia, triglycerides, HbA1c, and Orexin-A. For each regression, slope, intercept, 95% confidence intervals (CIs), coefficient of determination (*R* ^2^), and significance (F-statistics and *p*-value) were reported. A significant regression slope (*p* < 0.05) was interpreted as evidence of a statistically meaningful association between variables.

## Results

Statistically significant reductions were observed in all measured parameters (Table 1). The magnitude of these changes, expressed as Cohen’s *d*, indicated large effect sizes for all primary outcomes (ranging from 0.89 to 2.15), confirming the robustness and clinical relevance of the observed improvements. Specifically, serum AdoMet concentrations decreased markedly from 536.54 ± 248.91 pg/mL at T0 to 130.21 ± 88.03 pg/mL at T1 (p < 0.001). Body weight was significantly reduced from 93.95 ± 15.12 kg to 76.59 ± 14.53 kg (p < 0.001), with a corresponding decrease in BMI from 31.65 ± 4.43 kg/m^2^ to 26.47 ± 3.66 kg/m^2^ (p < 0.001). Visceral adipose tissue (VAT) decreased significantly from 1769.80 ± 751.11 g to 902.76 ± 544.97 g (p < 0.001). In terms of metabolic parameters, total cholesterol levels dropped from 241.28 ± 29.69 mg/dL to 184.38 ± 19.30 mg/dL (p < 0.001), glycemia from 111.76 ± 7.97 mg/dL to 87.04 ± 9.52 mg/dL (p < 0.001), and triglycerides from 176.21 ± 60.02 mg/dL to 79.57 ± 32.90 mg/dL (p < 0.001). A modest but statistically significant reduction was also observed in HbA1c, which decreased from 5.76% ± 0.42% to 5.53% ± 0.28% (p < 0.05). Finally, circulating levels of Orexin-A showed a significant decline from 3085.79 ± 87.25 pg/mL at T0 to 2862.49 ± 68.44 pg/mL at T1 (p < 0.001).

**FIGURE 1:**
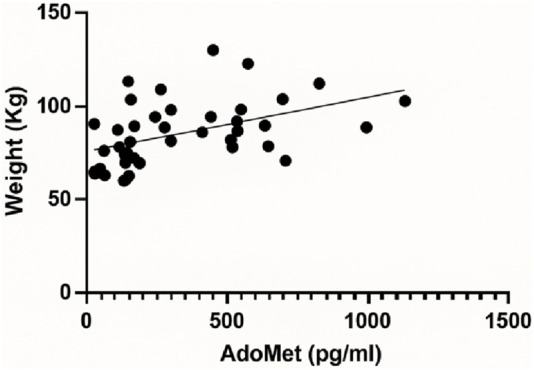
Linear regression analysis showing the relationship between serum AdoMet levels (pg/mL) and body weight (kg).

A linear regression analysis was performed to evaluate the relationship between AdoMet levels (pg/mL) and body weight (kg). The analysis revealed a statistically significant positive association between the two variables. The best-fit regression line was characterized by a slope of 0.02881 (±0.008655), with a y-intercept of 75.67 (±3.729). The 95% confidence interval for the slope ranged from 0.01132 to 0.04630, indicating that the slope was significantly different from zero (F(1, 40) = 11.08, p = 0.0019). The coefficient of determination (*R* ^2^) was 0.2169 (Figure 1).

**FIGURE 2:**
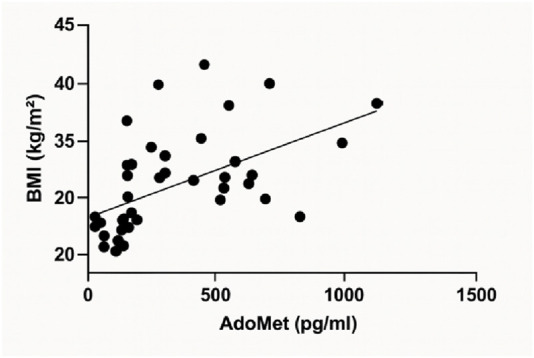
Linear regression analysis illustrating the relationship between serum AdoMet levels (pg/mL) and Body Mass Index (BMI, kg/m^2^).

A linear regression analysis was conducted to assess the relationship between serum AdoMet concentrations (pg/mL) and Body Mass Index (BMI, kg/m^2^). The analysis demonstrated a statistically significant positive correlation. The best-fit regression line was defined by a slope of 0.008556 (±0.002392) and a y-intercept of 26.21 (±1.031). The 95% confidence interval for the slope ranged from 0.003721 to 0.01339, indicating that the slope was significantly different from zero (F(1, 40) = 12.79, p = 0.0009). The coefficient of determination (*R* ^2^) was 0.2423 (Figure 2).

**FIGURE 3:**
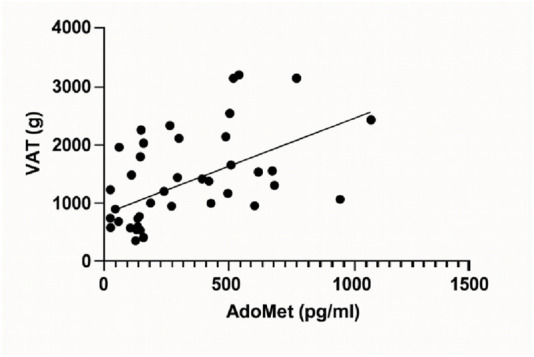
Linear regression analysis depicting the relationship between serum AdoMet levels (pg/mL) and visceral adipose tissue (VAT, g).

A linear regression analysis was conducted to examine the relationship between serum AdoMet levels (pg/mL) and visceral adipose tissue (VAT, g). The analysis revealed a statistically significant positive association. The best-fit regression line was characterized by a slope of 1.462 (±0.3838) and a y-intercept of 848.9 (±165.4). The 95% confidence interval for the slope ranged from 0.6863 to 2.238, confirming that the slope was significantly different from zero (F(1, 40) = 14.51, p = 0.0005). The coefficient of determination (*R* ^2^) was 0.2662 (Figure 3).

**FIGURE 4:**
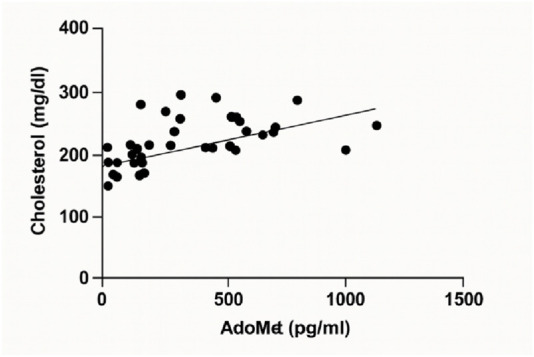
Linear regression analysis illustrating the relationship between serum AdoMet levels (pg/mL) and total cholesterol (mg/dL).

A linear regression analysis was performed to investigate the relationship between serum AdoMet levels (pg/mL) and total cholesterol concentrations (mg/dL). The analysis revealed a statistically significant positive association. The best-fit regression line had a slope of 0.06924 (±0.01877) and a y-intercept of 189.7 (±8.088). The 95% confidence interval for the slope ranged from 0.03130 to 0.1072, indicating that the slope was significantly different from zero (F(1, 40) = 13.60, p = 0.0007). The coefficient of determination (*R* ^2^) was 0.2538 (Figure 4).

**FIGURE 5:**
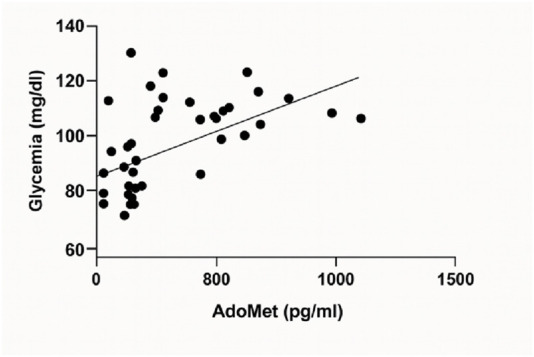
Linear regression analysis showing the relationship between serum AdoMet levels (pg/mL) and fasting glycemia (mg/dL).

A linear regression analysis was carried out to evaluate the relationship between serum AdoMet levels (pg/mL) and fasting glycemia (mg/dL). The analysis revealed a statistically significant positive association. The best-fit regression line had a slope of 0.02836 (±0.007470) and a y-intercept of 89.95 (±3.218). The 95% confidence interval for the slope ranged from 0.01327 to 0.04346, indicating that the slope was significantly different from zero (F(1, 40) = 14.42, p = 0.0005). The coefficient of determination (*R* ^2^) was 0.2650 (Figure 5).

**FIGURE 6:**
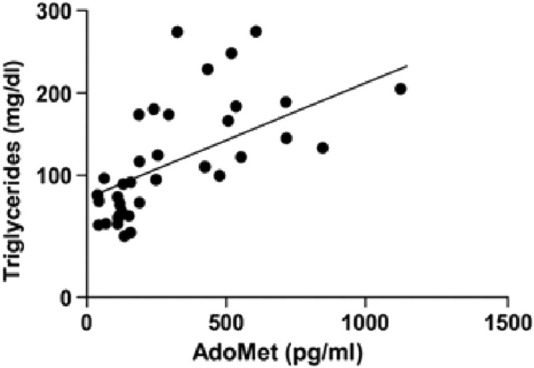
Linear regression analysis illustrating the relationship between serum AdoMet levels (pg/mL) and triglyceride concentrations (mg/dL).

A linear regression analysis was conducted to investigate the association between serum AdoMet levels (pg/mL) and triglyceride concentrations (mg/dL). The analysis revealed a statistically significant positive correlation. The best-fit regression line had a slope of 0.1287 (±0.03340) and a y-intercept of 84.89 (±14.39). The 95% confidence interval for the slope ranged from 0.06117 to 0.1962, indicating that the slope was significantly different from zero (F(1, 40) = 14.84, p = 0.0004). The coefficient of determination (*R* ^2^) was 0.2706 (Figure 6).

**FIGURE 7:**
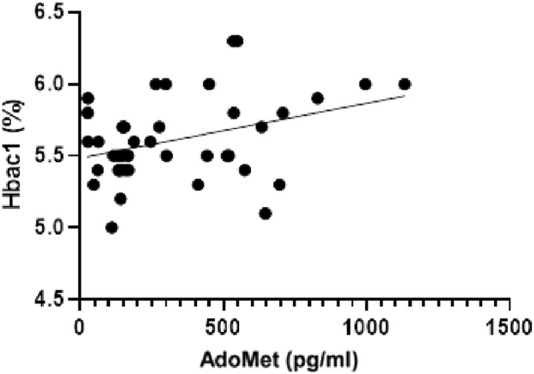
Linear regression analysis showing the relationship between serum AdoMet levels (pg/mL) and glycated hemoglobin (HbA1c, %).

A linear regression analysis was performed to explore the association between serum AdoMet levels (pg/mL) and glycated hemoglobin (HbA1c, %). The analysis revealed a statistically significant, though weak, positive correlation. The best-fit regression line was characterized by a slope of 0.0003820 (±0.0001566) and a y-intercept of 5.485 (±0.06746). The 95% confidence interval for the slope ranged from 6.549 × 10^−5^ to 0.0006984, indicating that the slope was significantly different from zero (F(1, 40) = 5.950, p = 0.0192). The coefficient of determination (*R* ^2^) was 0.1295 (Figure 7).

**FIGURE 8:**
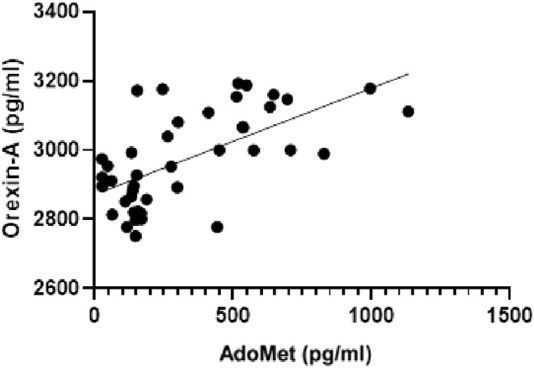
Linear regression analysis illustrating the relationship between serum AdoMet levels (pg/mL) and Orexin-A concentrations (pg/mL).

A linear regression analysis was conducted to evaluate the relationship between serum AdoMet levels (pg/mL) and Orexin-A concentrations (pg/mL). The analysis revealed a statistically significant positive correlation. The best-fit regression line was defined by a slope of 0.3077 (±0.06151) and a y-intercept of 2872 (±26.50). The 95% confidence interval for the slope ranged from 0.1834 to 0.4320, confirming that the slope was significantly different from zero (F(1, 40) = 25.02, p < 0.0001). The coefficient of determination (*R* ^2^) was 0.3848 (Figure 8).

**FIGURE 9:**
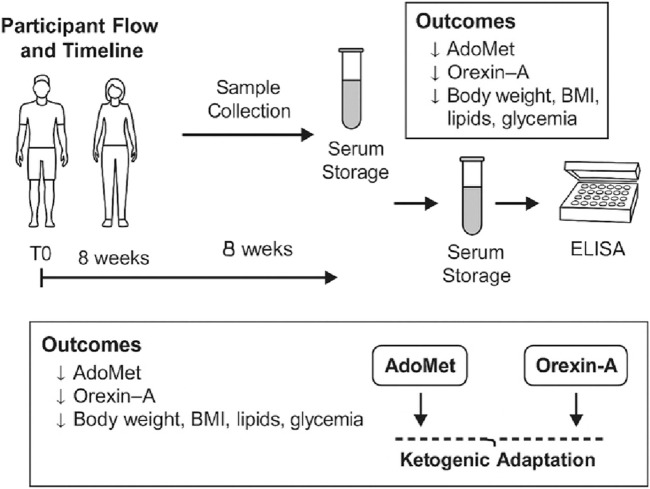
Schematic overview of study design, sample workflow, and main findings. The diagram illustrates the 8-week ketogenic dietary intervention and associated analyses. Participants underwent baseline assessment (T0), followed by 8 weeks of ketogenic nutrition and post-intervention evaluation (T1). Fasting blood samples were collected at both time points, aliquoted, stored at −80 °C, and analyzed by ELISA for S-adenosylmethionine (AdoMet) and Orexin-A concentrations. The main outcomes included significant reductions in circulating AdoMet, Orexin-A, body weight, BMI, lipid profile, and glycemia. The lower panel summarizes the hypothesized interaction between AdoMet and Orexin-A signaling during ketogenic adaptation, suggesting an integrated methylation-neuropeptide response contributing to improved metabolic homeostasis.

Taken together, the results indicate that the intervention led to a significant improvement across a wide range of anthropometric and metabolic parameters. Serum AdoMet levels decreased markedly and were accompanied by substantial reductions in body weight, BMI, visceral adipose tissue, total cholesterol, fasting glycemia, triglycerides, HbA1c, and Orexin-A. These changes were all statistically significant, highlighting the metabolic impact of the intervention (Figure 9).

Furthermore, linear regression analyses revealed that AdoMet levels were positively associated with several key metabolic indicators. Moderate but significant correlations were observed with body weight, BMI, visceral adipose tissue, total cholesterol, fasting glycemia, and triglycerides, suggesting that higher AdoMet concentrations are linked to less favorable metabolic profiles. A weaker yet significant association was also found with HbA1c. Notably, the strongest relationship emerged between AdoMet and Orexin-A levels, indicating a potential interaction between methylation pathways and neuropeptide signaling.

These findings suggest that circulating AdoMet may serve as a sensitive biomarker of metabolic status, reflecting not only changes in body composition and lipid/glucose metabolism, but also broader neuroendocrine regulation. The consistent associations observed reinforce the biological relevance of AdoMet in metabolic homeostasis and warrant further investigation.

## Discussion

The present study demonstrates that a structured intervention led to significant improvements in anthropometric and metabolic parameters, including substantial reductions in body weight, BMI, visceral adipose tissue, total cholesterol, fasting glycemia, triglycerides, HbA1c, and circulating levels of both AdoMet and orexin-A. The strong and positive correlation observed between AdoMet and orexin-A (*R* ^2^ = 0.3848, p < 0.0001) provides novel insight into the potential interplay between methylation pathways and neuropeptide signaling in the context of metabolic regulation.

AdoMet is a central methyl donor in one-carbon metabolism, influencing epigenetic regulation, lipid and glucose homeostasis, and inflammatory processes. Moreover, over the past 2 decades the antiproliferative properties of AdoMet have recently been highlighted by focusing on the identification of biological mechanisms and on the exploration of the signal transduction pathways connected to the chemopreventive activities of the sulfonium compound (Mosca et al., 2020; Mosca et al., 2024). In physiological condition, AdoMet reduction following the intervention aligns with the improvements observed in multiple metabolic parameters, suggesting that circulating AdoMet may be sensitive to changes in overall metabolic health. Orexin-A, a hypothalamic neuropeptide involved in energy expenditure, feeding behavior, and arousal, has also been implicated in glucose utilization and lipid oxidation. The parallel decline in both AdoMet and orexin-A, coupled with their strong association, raises the hypothesis that these two mediators may be functionally linked within an integrated neuro-metabolic network. It has been reported that AdoMet modulate hepatic β-oxidation and ATP synthesis playing a key physiological role as a metabolic sensor of nutrition, particularly in the hepatic fasting adaptive response (Capelo-Diz et al., 2023; Gu et al., 2017; Tang et al., 2022).

The ketogenic diet—characterized by low carbohydrate intake, moderate protein, and high fat—induces metabolic adaptations that increase ketone body production while influencing orexinergic signaling and methylation capacity. Ketone bodies such as β-hydroxybutyrate (BHB) have been shown to modulate histone acetylation and inflammatory responses, potentially interacting with AdoMet -dependent methylation pathways (Gurusamy et al., 2025). Furthermore, ketone-induced activation of orexin neurons has been linked to improved metabolic flexibility and energy balance. While our study did not directly implement a ketogenic diet, the metabolic profile observed—marked reductions in adiposity, improved lipid/glucose metabolism, and modulation of orexin-A—mirrors adaptations often reported under ketogenic conditions (Milbank and Lopez, 2019; Adeghate et al., 2020). The reduction in Orexin-A observed after the intervention may reflect a compensatory adjustment toward improved metabolic efficiency rather than diminished arousal or energy expenditure. Orexin neurons are highly responsive to nutrient availability and metabolic substrates; during ketosis, enhanced mitochondrial efficiency and lipid oxidation may reduce the energetic demand for orexin-mediated arousal signaling (Milbank and Lopez, 2019; Adeghate et al., 2020). Thus, decreased Orexin-A may signify an adaptive downregulation in a context of improved metabolic homeostasis, consistent with a shift toward more efficient substrate utilization.

The positive association between AdoMet and orexin-A suggests that methylation status may influence hypothalamic orexinergic tone, or vice versa, through shared nutrient-sensing and energy-regulatory mechanisms (Iacobini et al., 2022). One possible link is *via* mitochondrial function, which is a common regulatory target for both methyl donors and orexin signaling (Wachsmuth et al., 2022). Alternatively, reductions in AdoMet and orexin-A could both reflect systemic improvements in insulin sensitivity and lipid handling, given the consistent correlations of AdoMet with total cholesterol, triglycerides, and glycemia in our dataset.

From a translational perspective, the combined measurement of AdoMet and orexin-A may provide a dual biomarker approach for assessing metabolic health and responsiveness to dietary or lifestyle interventions, particularly those mimicking ketogenic physiology (Gu et al., 2017; Liu et al., 2020). However, the directionality and causality of their relationship remain to be determined, and future studies should include mechanistic analyses—potentially integrating dietary manipulations such as controlled ketogenic feeding, dose-response studies, and evaluation of gene expression related to methylation enzymes and orexin receptors (Ronn et al., 2023; Brown et al., 2018; Pietzke et al., 2020).

In addition, given the established roles of AdoMet and Orexin-A in neuro-metabolic homeostasis, the observed modulation may also have relevance for conditions such as obesity, insulin resistance, type 2 diabetes, and neurodegenerative diseases characterized by metabolic dysregulation (Liu Y et al., 2024; Wang et al., 2022). Both molecules influence neuronal energy metabolism and synaptic plasticity, suggesting that dietary modulation of AdoMet and Orexin-A could represent a therapeutic target in disorders involving impaired mitochondrial or methylation-dependent signaling.”

In summary, our findings highlight a novel link between AdoMet and orexin-A in the context of metabolic improvement. This association reinforces the concept that metabolic homeostasis is orchestrated by a dynamic interplay between epigenetic regulation and neuroendocrine signaling—both of which can be modulated by dietary and lifestyle factors, including ketogenic-like interventions.

Although the findings highlight robust associations between AdoMet, Orexin-A, and metabolic parameters, the absence of a parallel control group following a standard diet limits the ability to establish causal relationships. Future studies including randomized controlled designs are warranted to confirm these associations and elucidate underlying mechanisms.

Recent advances in precision nutrition highlight that individual variability in response to ketogenic diets is influenced by genetic polymorphisms, epigenetic regulation, microbiome composition, and baseline metabolic status (Liu Y et al., 2024). Such factors may modulate both AdoMet-dependent methylation and orexinergic signaling, resulting in heterogeneous metabolic outcomes across populations. Future personalized approaches integrating genomic and metabolomic profiling may refine the predictive value of AdoMet and Orexin-A as biomarkers of dietary responsiveness.

This study has several limitations, including the small sample size, lack of a control group, and absence of mechanistic analyses of methylation or orexin receptor signaling. Moreover, the relatively short intervention period (8 weeks) precludes long-term extrapolation. Despite these limitations, the consistent trends observed suggest biologically meaningful interactions warranting validation in larger, randomized cohorts.

## Data Availability

The original contributions presented in the study are included in the article/Supplementary Material, further inquiries can be directed to the corresponding author.
