# Supplementary material for: Ketogenic diet–induced changes in methylation status and neuropeptide signaling: relationships between S-adenosylmethionine (AdoMet), orexin-A, and metabolic health
Source: Front Physiol. 2025 Nov 4;16:1719549. doi: 10.3389/fphys.2025.1719549 (PMC12623159; doi:10.3389/fphys.2025.1719549)
Supplement: Supplementary file 1 [file Image1.pdf]

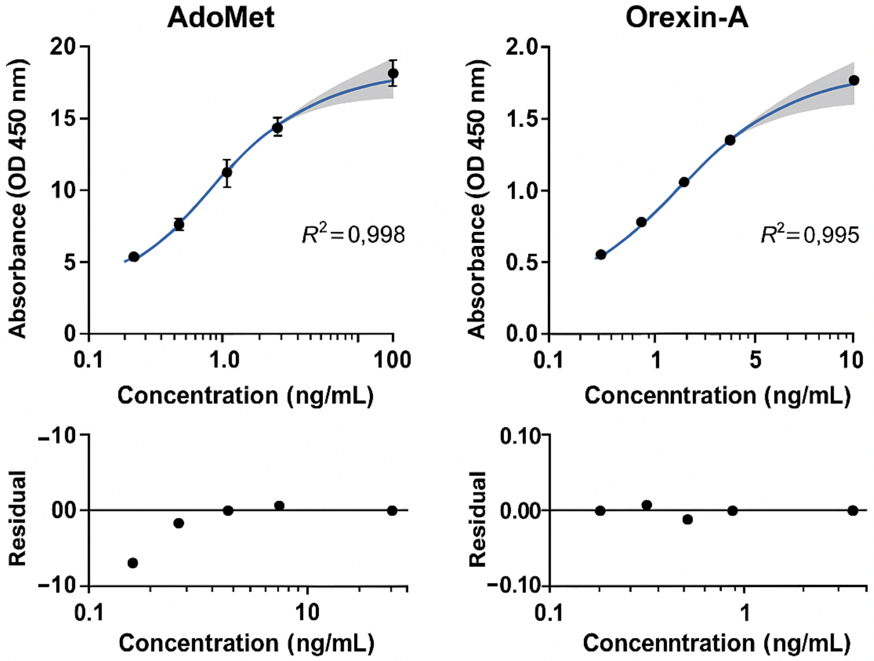

Supplementary Figure S1. Representative calibration curves for AdoMet and Orexin-A ELISAs, Standard curves were fitted using a five-parameter logistic (S-PL) regression model (R^2 > 0,99 across all assays). Lower panels display residual plots showing random distribution around zero, confirming goodness-of-fit and absence of error.
